# Supplementary material for: Experimental demonstration of tethered gene drive systems for confined population modification or suppression
Source: BMC Biol. 2022 May 24;20:119. doi: 10.1186/s12915-022-01292-5 (PMC9128227; doi:10.1186/s12915-022-01292-5)
Supplement: Supplementary file 1 — Additional file 1: List of plasmid construction primers and methods. Figure S1. Tethered drive mechanism. Figure S2. Relations between genotypes in the TARE drive. Figure S3. Egg-to-pupae viability for haplolethal homing drive. Figure S4. Population sizes for cage study. Table S1. Comparison of different gene drive types. Table S2. Combined maximum likelihood parameter estimates from cage populations. [file 12915_2022_1292_MOESM1_ESM.pdf]

Experimental demonstration of tethered gene drive systems for confined population modification or suppression

Matthew Metzloff[1,2], Emily Yang[1,2], Sumit Dhole[3], Andrew G. Clark[1,2], Philipp W. Messer[1], Jackson Champer[1,2,4*]

[1]Department of Computational Biology, Cornell University, Ithaca, NY 14853
[2]Department of Molecular Biology and Genetics, Cornell University, Ithaca, NY 14853
[3]Department of Entomology and Plant Pathology, North Carolina State University, Raleigh, North Carolina 27695
[4]Current Address: Center for Bioinformatics, School of Life Sciences, Peking-Tsinghua Center for Life Sciences, Peking University, Beijing, China 100871

*Corresponding author: JC (jchamper@pku.edu.cn)

SUPPLEMENTARY INFORMATION

The following table shows the DNA fragments used for Gibson Assembly of the plasmid. PCR products are shown with the oligonucleotide primer pair used, and plasmid digest is shown with the restriction enzymes used.

**TAREhNU2G**	*Template*	*Oligo/Enzyme 1*	*Oligo/Enzyme 2*
PCR Product	BHDaaN	SV40_U6_F	NosCas9_1_R
PCR Product	BHDaaN	Cas9_2_F	Nos3_3x_R
Plasmid Digest	EGDh2	HindIII	MluI

Construction primers

```
Cas9_2_F: AAACAGCTCAAGAGGCGCC
Nos3_3x_R: TTCAATTAGAGCTAATTCAATTAGGATCCAAGCTTTCCTTCCTGGCCCTTTTCGA
NosCas9_1_R: TCCTGTATATCGGCGCCTCTT
SV40_U6_F: GGAGCAATCACAGGTGAGCAAAAAAACGCGTTAAGATACATTGATGAGTTTGGACAAACC
```

Sequencing primers

```
EGFP_S_F: AGCGCACCATCTTCTTCAAGG
h3utr_S_F: AAGGACCTTCATCAGACGCAC
hCut_S_F: CCAAATTGGAAAAGGCCGACA
hCut_S_R: AACATGGGTTGCTGTTGTGC
```

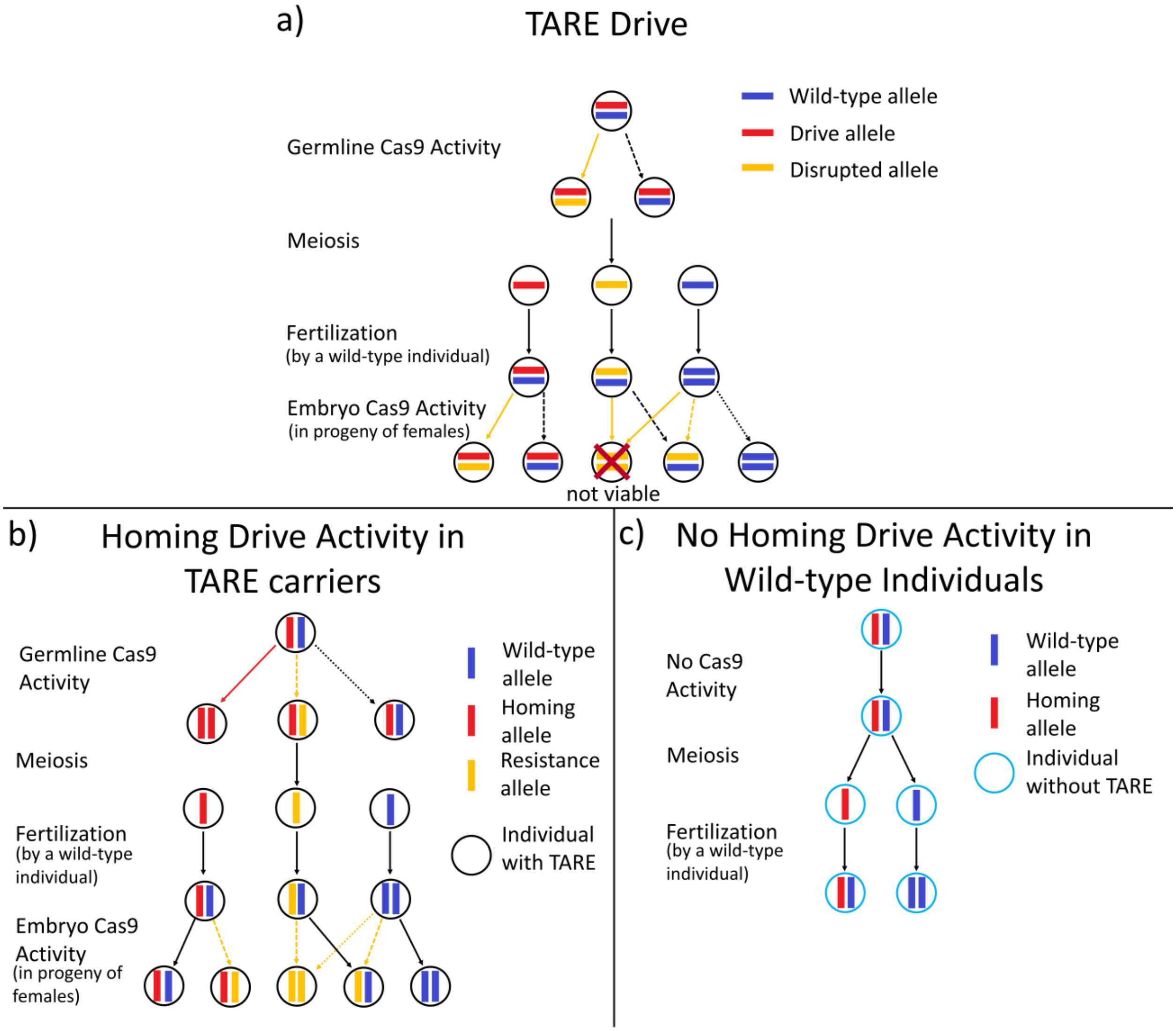

Figure S1 Tethered drive mechanism. a) A regionally confined TARE drive is first released. Cas9 activity occurs in the germline of drive-carrying individuals, disrupting wild-type alleles. Then, embryo Cas9 activity occurs in the progeny of females. Individuals with two disrupted alleles are not viable. Eventually, the TARE drive will spread in the target population. Common events are indicated with solid arrows, and dashed arrows indicate less common events. **b)** A homing drive is released into the population of individuals with the TARE drive (or another confined drive with Cas9). Cas9 activity leads to drive conversion by homology-directed repair or to resistance alleles (which can be removed in a manner dependent on the homing drive's target gene). **c)** Homing drive activity cannot occur in individuals that do not express Cas9, thus confining it to the population that contains the TARE drive.

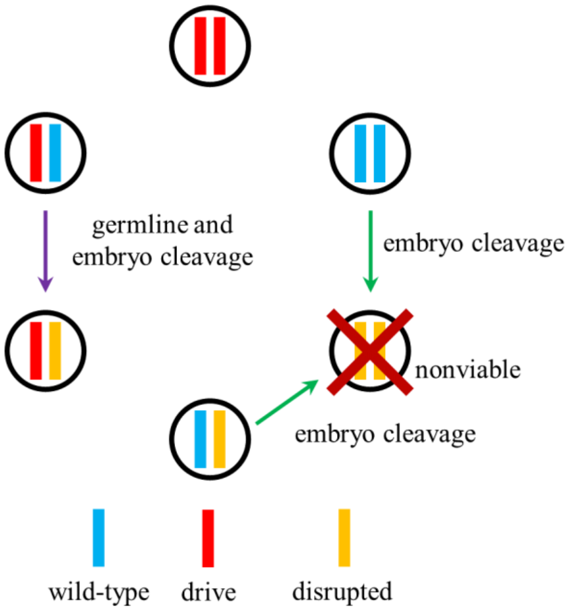

Figure S2 Relations between genotypes in the TARE drive. There are six possible genotypes for the TARE drive, representing combinations between drive, disrupted, and wild-type alleles. Drive/wild-type germline cells are usually converted to drive/disrupted heterozygotes in both males and females. Additionally, any wild-type allele in any genotype can be converted to a disrupted allele due to embryo Cas9 activity if the mother carried a drive allele. Individuals that are homozygous for disrupted alleles are nonviable. Thus, wild-type alleles in a population will tend to be converted to disrupted alleles, which are often nonviable, thus increasing the frequency of drive alleles in the population.

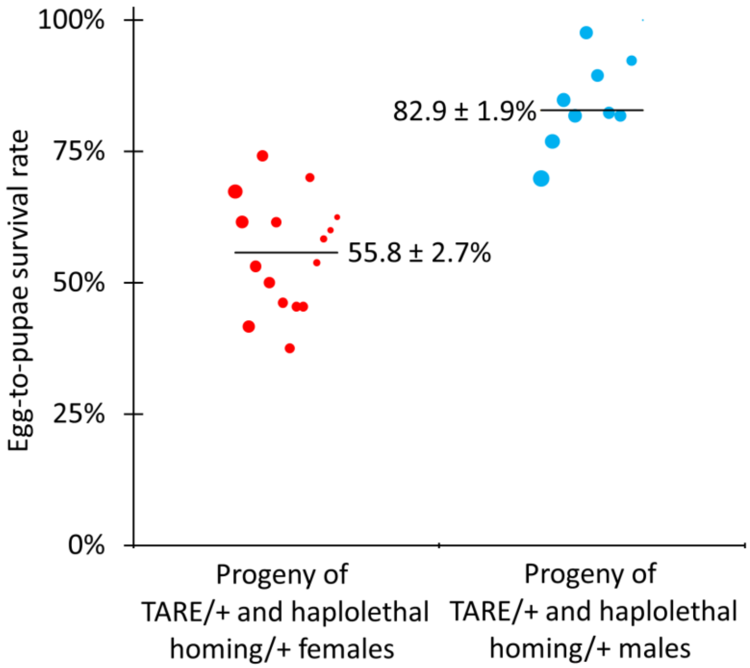

Figure S3 Egg-to-pupae viability for haplolethal homing drive. Individuals heterozygous for the TARE drive and the haplolethal homing drive were crossed with w^{1118} individuals, and eggs and pupae were counted as well as eclosed adults. The size of the dots is proportional to the number of eggs from a single female. Rate estimates and SEM are indicated.

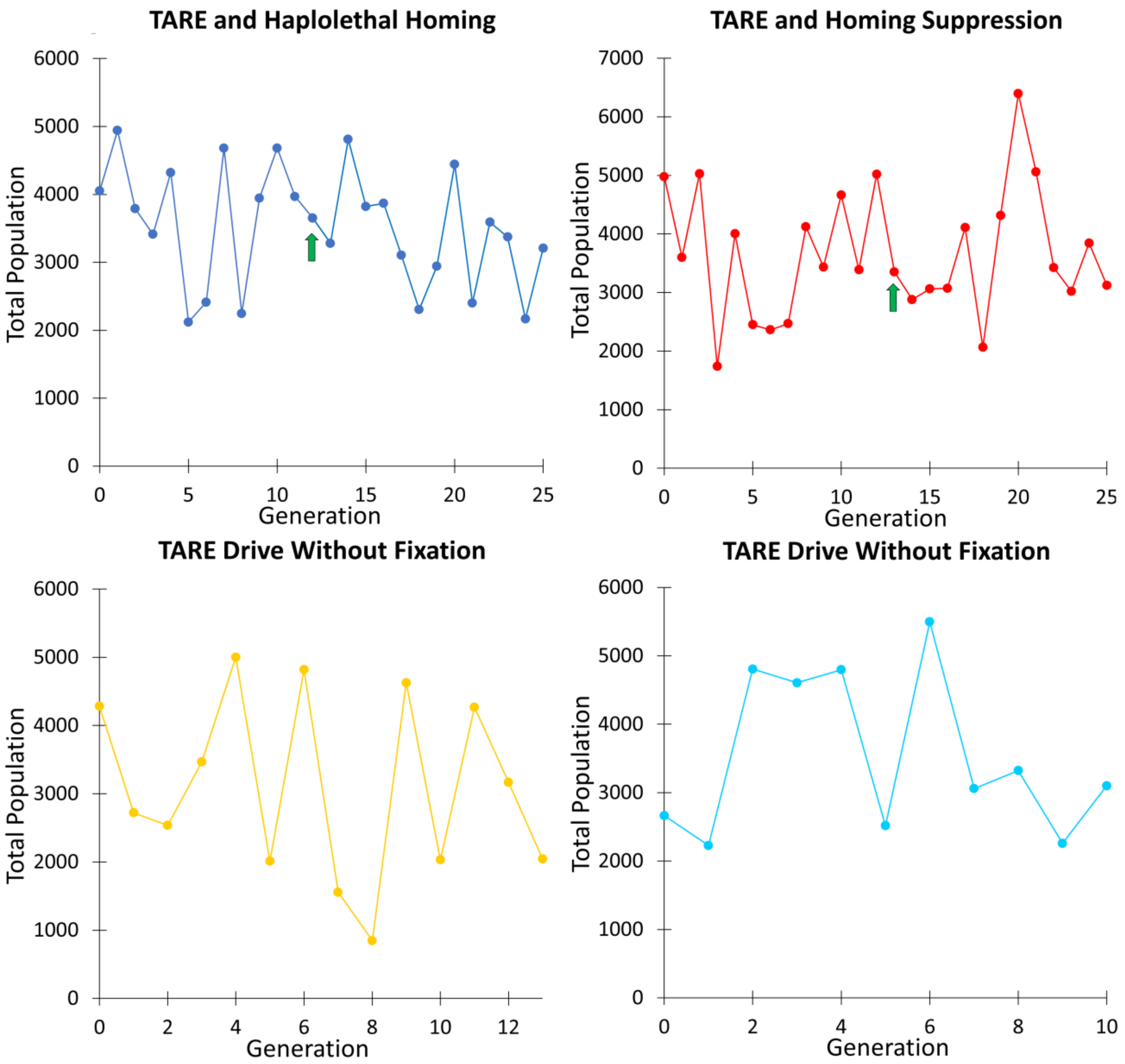

Figure S4 Population sizes for cage study. The population is shown for each generation. Arrows indicate generations where homing drives were released in the first two cages.

Table S1. Comparison of different gene drive types

Drive type	High power suppression?	Threshold	Target gene	Preferred cas9 promoter	Engineering difficulty	Specific challenges
**Homing drive**	yes	zero	essential	germline	low	requires high drive conversion
**Split homing drive**	no	self-limiting	essential	germline	low	requires high drive conversion
**Daisy drive**	yes*	eventually self-limiting	multiple essential	germline	moderate?	multiple targets
**Y-linked X-shredder**	yes	zero	X-chromosome	germline/ embryo	high	Y chromosome engineering
**Killer-rescue**	no	self-limiting	variable	variable	moderate	variable mechanism
**TARE**	no	low	essential	any	low	none
**2-locus TARE**	no	moderate	two essential	any	low?	two alleles
**1-locus 2-drive TARE**	no	high	two essential	any	low?	two alleles
**TADE**	yes	low-moderate	haplolethal	germline	moderate?	haplolethal target
**TADE underdominance**	yes	moderate-high	haplolethal	germline/ embryo	moderate?	haplolethal target
Wolbachia	no	moderate	N/A	N/A	low	complex organism
**Tethered drive system**	yes	varies**	as above	germline	low	requires high drive conversion

N/A - not applicable. *High power suppression until some drive elements are no longer supported by earlier elements. **Tethered drives have variable confinement based on their cas9 containing element. "?" indicates that engineering difficulty is an estimate. Specific challenges can be highly variable based on the target species.

Table S2. Combined maximum likelihood parameter estimates from cage populations

Log-likelihood shows a relative probability (higher values indicate a better model fit)
AICc: Akaike information criterion, corrected (low values indicate a better match of the model without overfitting)
Effective population size refers to the percent of census size, which varies with each generation (the average was 3491)
Fitness values are for drive homozygotes (with multiplicative fitness per allele). A value of 1 is equivalent to wild-type fitness, and "1" indicates that the parameter was fixed

**Fitness cost model**	**Log-likelihood**	**AICc**	**Effective population size**	**Mating fitness**	**Fecundity fitness**	**Viability fitness**
**None**	96.5	-191.0	3.02%	1	1	1
**All**	100.4	-191.9	3.68%	0.943	0.846	0.962
**All with mating = fecundity**	100.4	-194.3	3.58%	0.867	0.867	1.00
**Mating and fecundity**	100.8	-194.9	3.64%	1.00	0.754	1
**Mating and viability**	100.3	-194.0	3.56%	0.741	1	1.00
**Fecundity and viability**	100.8	-194.9	3.64%	1	0.7539	1.00
**Mating**	100.3	***-196.3***	3.57%	0.7322	1	1
**Fecundity**	100.8	***-197.2***	3.64%	1	0.754	1
**Viability**	100.0	***-195.7***	3.52%	1	1	0.853
**Mating = fecundity**	100.4	***-196.6***	3.59%	0.867	0.867	1
